# Study protocol to investigate the effect of a lifestyle intervention on body weight, psychological health status and risk factors associated with disease recurrence in women recovering from breast cancer treatment [ISRCTN08045231]

**DOI:** 10.1186/1471-2407-6-35

**Published:** 2006-02-09

**Authors:** John M Saxton, Amanda Daley, Nicola Woodroofe, Robert Coleman, Hilary Powers, Nanette Mutrie, Vanessa Siddall, Helen Crank

**Affiliations:** 1 Centre for Sport and Exercise Science, Sheffield Hallam University, Sheffield, UK; 2 Department of Primary Care and General Practice, University of Birmingham, UK; 3 Biomedical Research Centre, Sheffield Hallam University, Sheffield, UK; 4 Academic Unit of Clinical Oncology, University of Sheffield, Weston Park Hospital, Sheffield, UK; 5 Centre for Human Nutrition, University of Sheffield, UK; 6 Department of Sport, Culture and the Arts, Strathclyde University (Jordanhill Campus), Glasgow, UK

## Abstract

**Background:**

Breast cancer survivors often encounter physiological and psychological problems related to their diagnosis and treatment that can influence long-term prognosis. The aim of this research is to investigate the effects of a lifestyle intervention on body weight and psychological well-being in women recovering from breast cancer treatment, and to determine the relationship between changes in these variables and biomarkers associated with disease recurrence and survival.

**Methods/design:**

Following ethical approval, a total of 100 patients will be randomly assigned to a lifestyle intervention (incorporating dietary energy restriction in conjunction with aerobic exercise training) or normal care control group. Patients randomised to the dietary and exercise intervention will be given individualised healthy eating dietary advice and written information and attend moderate intensity aerobic exercise sessions on three to five days per week for a period of 24 weeks. The aim of this strategy is to induce a steady weight loss of up to 0.5 Kg each week. In addition, the overall quality of the diet will be examined with a view to (i) reducing the dietary intake of fat to ~25% of the total calories, (ii) eating at least 5 portions of fruit and vegetables a day, (iii) increasing the intake of fibre and reducing refined carbohydrates, and (iv) taking moderate amounts of alcohol. Outcome measures will include body weight and body composition, psychological health status (stress and depression), cardiorespiratory fitness and quality of life. In addition, biomarkers associated with disease recurrence, including stress hormones, estrogen status, inflammatory markers and indices of innate and adaptive immune function will be monitored.

**Discussion:**

This research will provide valuable information on the effectiveness of a practical, easily implemented lifestyle intervention for evoking positive effects on body weight and psychological well-being, two important factors that can influence long-term prognosis in breast cancer survivors. However, the added value of the study is that it will also evaluate the effects of the lifestyle intervention on a range of biomarkers associated with disease recurrence and survival. Considered together, the results should improve our understanding of the potential role that lifestyle-modifiable factors could play in saving or prolonging lives.

## Background

Although the number of women who survive breast cancer is increasing, survivors often encounter physiological and psychological problems related to their diagnosis and treatment that can influence long-term prognosis. A gain in body weight [1-6] and psychosocial distress [7-12] are two commonly encountered adverse responses to breast cancer diagnosis and treatment that can have a negative impact on quality of life and survival. Lifestyle strategies that can attenuate the negative effects of such physical and mental health outcomes after breast cancer diagnosis could further improve the quality of life and long-term prognosis of breast cancer survivors.

Up to 60% of women diagnosed with breast cancer experience an increase in body weight associated with chemotherapy and treatment-related menopause [6] and there is evidence that heavier women and women who gain weight after diagnosis have an increased risk of disease recurrence and death compared to normal weight women [1-5]. Women with a high body mass index (BMI) have double the risk of 5-year recurrence and a 60% increased risk of death over 10 years in comparison to normal-weight women [5]. Poorer survival in heavier patients could be due to higher levels of tumor-promoting hormones, consequent to an increased adiposity [13]. There is evidence that elevated estrogen levels influence the risk of breast cancer in postmenopausal women [14-16] and it was recently shown that overweight postmenopausal breast cancer patients have higher estrogen and testosterone levels than normal-weight patients [13]. This led to the suggestion that circulating estrogen levels could be a useful biomarker for weight loss intervention studies [13].

Following breast cancer diagnosis or treatment, four in ten women also experience high levels of emotional distress, including depression and anxiety [7], which persists for prolonged periods of time, irrespective of the treatment outcome [8-12]. Studies have shown that psychosocial stressors such as depression, anxiety and emotional distress are associated with impaired immune function [17], and this could have a direct impact on cancer outcomes [18,19] and influence the risk of disease re-occurrence and death [20]. The adverse effect of psychosocial stressors on immune function is considered to be mediated by excess secretion of the stress hormone cortisol and the catecholamines [21,22]. Elevated cortisol and catecholamine levels evoked by psychological stressors can significantly influence immune function, including lymphocyte proliferation and natural killer (NK) cell activity [23-26]. Self-reported negative mood states have been associated with lower NK cell activity [27,28] and symptoms of depression have been linked with increased salivary cortisol [29] impaired lymphocyte proliferation [30,31] and reduced NK cell cytotoxicity against tumor cells [32-34]. In addition, studies have reported abnormal circadian rhythmicity of cortisol in breast cancer patients [35-39] and flattened or abnormal diurnal salivary cortisol rhythms have recently been associated with earlier mortality in breast cancer patients [38] and persistent fatigue in survivors 1–5 years after initial diagnosis [40].

Dietary energy restriction can reduce body weight and induce a positive effect on psychological well being in obese women and breast cancer survivors [41-44]. Weight loss interventions that reduce dietary intake of fat to 18–25% of the total calories can also evoke a significant reduction in serum estrogen levels in pre and postmenopausal women [45]. The positive effects of dietary energy restriction on these physical and emotional sequelae might be augmented by the implementation of adjunctive exercise therapy [46,47]. Regular physical activity can help to control body weight and is known to reduce the risk of breast cancer [48-53]. Recent evidence also suggests that it can halve the risk of death in breast cancer patients [54]. The precise mechanisms underpinning the positive effects of physical activity on breast cancer risk and survival are unknown, although physical activity has been associated with a lower concentration of circulating estrogen in some studies [55,56], as well as lower circulating levels of the inflammatory mediators, C-reactive protein (CRP) [57-59] and interleukin-6 (IL-6) [60,61]. Increased CRP levels are observed in overweight individuals [62,63] and have been linked with increased cancer risk [64,65] and elevated levels of cortisol in individuals subjected to psychological stress [66]. Depression enhances the production of IL-6 [67-69] and this proinflammatory cytokine is also secreted from adipocytes in proportion to body fat mass [60]. IL-6 induces CRP synthesis in the liver and can indirectly raise cortisol levels via stimulation of corticotropin-releasing hormone production [67]. Elevated estrogen levels may also increase CRP levels through a non-inflammatory mechanism [70].

Regular physical activity can also have a positive effect on psychological health status [71,72] and quality of life [73] in breast cancer survivors that could enhance immune function through normalisation of stress hormone levels. Although there is evidence for decreased cortisol levels [74,75] and changes in immune cell numbers and function after psychosocial interventions in breast cancer patients [76,77], only three studies have investigated the effects of exercise therapy on immune function in breast cancer survivors. Peters *et al*. [78,79] reported an increase in the percentage of NK cells and an improvement in NK cell function and monocyte phagocytic capacity that was accompanied by an increase in 'satisfaction of life' score after seven months of moderate exercise training, whereas Nieman *et al*. [80] did not observe any change in NK cell function following a shorter period of training. The latter study had only six subjects in the experimental (exercise therapy) group, and neither study measured stress hormone levels in patients. In a more recent study, Fairey *et al*. [81] showed that a 15 week programme of aerobic exercise training improved NK cell cytotoxic activity and peripheral blood lymphocyte function in postmenopausal breast cancer survivors. In sedentary, healthy individuals, regular moderate aerobic exercise has been shown to boost NK cell function [82] and decrease the incidence of upper respiratory tract infections in previously sedentary individuals [82-84]. In elderly females however, evidence for an improvement in immune cell function with aerobic exercise training is inconclusive, although highly trained elderly females exhibit superior resting NK cell function and T cell proliferation responses compared to age-matched sedentary controls [83]. The lack of intervention studies incorporating biomarkers of immune function in women recovering from breast cancer treatment is surprising, as restoration of lymphocyte and NK cell function after treatment is predictive of survival [28,85,86] and is independent of stage of disease and adjunctive treatment approach [86]. This suggests that lifestyle interventions which could have an impact on immune function are worth exploring, as they could have a significant influence on disease-free survival in breast cancer patients.

Previous studies have investigated the feasibility and efficacy of lifestyle interventions in breast cancer patients and obese women, in terms of recruitment, compliance and weight loss [42,43,87,88]. Some studies have also examined the effects of lifestyle interventions on aspects of psychological [41,47,71,72] and physiological [42,87,88] well-being. However, no previous studies have investigated the effects of a dietary and exercise intervention on a range of important prognostic breast cancer biomarkers that could be strongly influenced by weight loss and improved psychological well-being. Thus, the aim of the intended research is to examine the effects of a dietary and exercise intervention on body weight, indices of psychological well-being and biomarkers associated with disease recurrence and physiological health status in female breast cancer survivors. We hypothesize that the lifestyle intervention will evoke a reduction in body weight and an improvement in psychological well-being. Secondly, that improvements in blood markers of cancer risk and physiological health status (estrogen, CRP, IL-6), psychosocial stress (cortisol and catecholamines) and immune function (lymphocyte proliferation and NK cell function) will be associated with changes in body weight and psychological well-being induced by the lifestyle intervention. Considered together, the results of this study would provide valuable new information to improve our understanding of the role that lifestyle-modifiable factors can play in saving or prolonging the lives of women who have been treated for breast cancer.

## Research design and methods

### Overall aim of the study

To examine the effects of a practical lifestyle intervention that could be easily implemented by overweight or obese women (BMI > 25) who have undergone breast cancer treatment, on body weight, indices of psychological well-being, biomarkers associated with disease recurrence and physiological health status and quality of life.

### Primary aims

1. To examine the effects of a dietary and exercise intervention on body weight and body composition in overweight or obese women who have undergone breast cancer treatment.

2. To examine the effects of a dietary and exercise intervention on indices of psychological health status and biomarkers associated with disease recurrence in overweight or obese women who have undergone breast cancer treatment.

### Secondary aims

1. To examine the relationship between change in body weight and biomarkers associated with disease recurrence and physiological health status, including circulating levels of estrogen, CRP and IL-6.

2. To examine the relationship between changes psychological health status, circulating stress hormones and indices of immune function.

3. To examine the effects of the dietary and exercise intervention on broader quality of life dimensions.

### Patient recruitment

A total of 100 women (BMI > 25) who have undergone appropriate treatment for operable breast cancer within the past 3–18 months and are no longer undergoing chemotherapy or radiation therapy, will be recruited from the Cancer Research Centre, Weston Park Hospital, Sheffield University Hospitals NHS Trust. The incidence of breast cancer within the North Trent Regional Health Authority area is relatively high, with 850 cases per year and, on the basis of current rates of breast cancer survival in South Yorkshire and recruitment rate in our ongoing trial, we expect to recruit patients at the rate of approximately six per month for 16 months. Ethical approval has been obtained from the South Sheffield Research Ethics Committee and informed consent will be obtained from all participants before they enter the study.

### Randomisation

The minimisation technique [89] will be used to allocate the patients into one of two groups (i) lifestyle intervention group, incorporating moderate dietary energy restriction of 600 kcal per day in conjunction with aerobic exercise training on 3–5 days per week, or (ii) normal care control group. Minimisation will be used to balance the potentially confounding variables of chemotherapy and treatment with Tamoxifen, aromatase inhibitors or no hormone therapy across the groups. Randomisation will be performed by an independent researcher at the Clinical Trials Research Unit, University of Leeds. The intervention will last for 24 weeks, with outcome measures being assessed in the week before and the week after the intervention period and at the mid-intervention time point of 12 weeks. Patients would be recruited over 16 months, ensuring completion of the trial by 22 months and reporting of the data by 24 months.

### Details of power calculations and sample size

Body weight was chosen as the primary outcome variable for calculation of sample size. Utter *et al*. [88] reported a 8.1 kg reduction in body weight in obese women following a 12 week lifestyle intervention, incorporating moderate dietary energy restriction in conjunction with exercise. Body weight changed from a baseline ± SD level of 89.9 ± 11.7 to 81.8 ± 10.8 kg following the intervention. This amount of weight loss is associated with improved physical and psychological health in obese women [41,90]. On the basis of these data, and taking into account an expected patient drop-out of up to 10% (based on the drop-out rate in our ongoing trial with breast cancer survivors [91]), recruitment of 50 patients for each group will give us 90% power to detect a difference in body weight of 8 kg at the α level of 0.05.

### Patient inclusion criteria

a. Postmenopausal women (confirmed by plasma estradiol and gonadotrophin measures in all women aged <55) with a BMI > 25 and classified as disease stage I-III. In our experience [91], a high proportion of breast cancer patients (>80%) meet the BMI criteria and would be suitable for participation in the study.

b. Patients must have completed some form of breast cancer treatment at least three months, and not more than 18 months ago.

c. Patients on Tamoxifen and other endocrine treatments but not hormone replacement therapy (HRT) will be included.

d. Patients must be willing and able to attend supervised exercise sessions at least 3 times per week for a period of 24 weeks, with the intention of achieving an 80% minimum compliance target for attendance.

e. Patients must be an exercise pre-contemplator, contemplator or preparer as defined by the Transtheoretical Model [92].

### Patient exclusion criteria

a. Metastatic breast cancer patients and patients with inoperable or active loco-regional disease.

b. Patients following alternative/complementary diets or taking high dose antioxidant supplements.

c. Patients with a physical/psychiatric impairment that would seriously impair their physical mobility.

d. Patients who are currently suffering from severe nausea, anorexia or other diseases affecting health (e.g. arthritis and multiple sclerosis).

e. HRT is not commonly prescribed in women who are recovering from breast cancer treatment, but use of HRT or oral contraceptives within the past four months is an exclusion criteria.

f. Patients who are currently engaged in exercise (two or more times per week for at least 30 min per session during the previous 3 months).

### Supervised exercise

Patients randomised to the dietary and exercise intervention will attend moderate intensity aerobic exercise sessions on three to five days per week for a period of 24 weeks under the supervision of an exercise scientist. To account for differences in daily patterns of fatigue and to minimise attrition, patients will be offered different times to chose from including week-ends. Patients will be encouraged to attend five supervised sessions each week, and must try to attend at least three of the sessions. Patients not able to attend five supervised sessions will be counselled on how they can fit an extra 1–2 home/community-based exercise sessions into their weekly routine. All sessions of physical activity will be recorded in a physical activity log and portable pedometers (Omron Healthcare, UK) will be used to determine the number of steps/distance walked each day. Supervised exercise will be performed in groups of up to eight participants in an exercise room that contains a variety of aerobic exercise equipment. Each session will comprise a 10 minute warm-up period (involving light aerobic exercise and gentle range of motion exercises), 30 min of aerobic exercise at an intensity of 70%–85% heart rate reserve, and a 10 min cool-down period involving lower intensity aerobic exercise and some light stretching. Patients will wear heart rate monitors throughout the exercise sessions and heart rate and ratings of perceived exertion [93] will be assessed at regular intervals. In accordance with the recommendations of Biddle *et al*. [94], patients will be offered a range of aerobic exercise modalities at these sessions (e.g. stepping, cycling, & walking/jogging) to promote enjoyment and to aid compliance to the programme. Patients will be encouraged to use their preferred exercise mode to strengthen the possibility of them maintaining a physically active lifestyle when the supervised exercise programme has been completed. The exercise therapy sessions will use a variety of cognitive-behavioural techniques for promoting exercise adherence as well as positive attitudes and experiences.

### Dietary energy restriction

Patients randomised to the dietary and exercise intervention will be given individualised healthy eating dietary advice and written information: 'Weight Loss On A Plate' (Scottish Dietetic Association). The focus of the verbal advice will be on reducing the patient's total daily calorie intake to 600 kcal below their calculated energy requirements. Individual energy requirements will be estimated from formulae of basal metabolic rate and physical activity level (Scottish Dietetic Association). The aim of this strategy is to induce a steady weight loss of up to 0.5 Kg each week. In addition, the overall quality of the diet will be examined with a view to (i) reducing the dietary intake of fat to ~25% of the total calories, (ii) eating at least 5 portions of fruit and vegetables a day, (iii) increasing the intake of fibre and reducing refined carbohydrates, and (iv) taking moderate amounts of alcohol. Patients will be required to complete a 3 day diet diary pre intervention and then weekly throughout the study. The diary will include one day from the weekend. The diaries from pre intervention and from weeks 6, 12, 18 and 24 will be analysed, looking specifically at macronutrients, using Dietmaster software (SDA Solutions, London, UK) computer dietary analysis package. Once a week, patients will meet with the research assistant who will discuss their individual diet diaries with them and identify ways which they can further improve their nutritional intake. Patients in the control group will be provided with a general healthy eating booklet 'Getting the Balance Right' (British Nutrition Foundation).

### Outcome measures

The intervention will last for 24 weeks, with all outcome variables being assessed in the week before and the week after the intervention period unless otherwise stated.

### Primary outcome measure

#### Body weight and body composition

Patients will be weighed to the nearest 0.05 kg and percentage body fat estimated using bio-electrical impedance (Bodystat 1500, Bodystat Ltd., UK). Waist and hip girths will be measured to the nearest 0.5 cm using an inelastic circumference tape. Body weight will be measured at weekly intervals in the intervention group, whereas bio-electrical impedance analysis and girth measurements will only be performed in the week before and after the intervention period and at the mid-intervention time point of 12 weeks.

### Other outcome measures

#### Psychological indices, cardiorespiratory fitness and quality of life

##### Psychological stress

Psychological stress will be assessed using the Perceived Stress Scale (PSS) [95]. The PSS is one of the most widely used psychological instrument for measuring the perception of stress. It measures the degree to which situations in an individual's life are appraised as stressful. The items evaluate how unpredictable, uncontrollable, and overloaded respondents find their lives. The scale also includes a number of direct queries about current levels of experienced stress. Psychological stress will be assessed in the week before and after the intervention period and at the mid-intervention time point of 12 weeks.

##### Depression

Depression will be assessed using the Beck Depression Inventory II (BDI-II) [96]. The BDI is a 21-item test which measures the presence and degree of depression in respondents. It assesses specific symptoms or attitudes which appear to be specific to depressed patients, and which are consistent with descriptions of the depression contained in the psychiatric literature. Depression will be assessed in the week before and after the intervention period and at the mid-intervention time point of 12 weeks.

##### Cardiorespiratory fitness

Cardiorespiratory fitness will be assessed using a single-stage submaximal treadmill walking test [97]. Improved cardiorespiratory fitness in breast cancer patients following a 16 week exercise intervention has previously been demonstrated using this treadmill test [98]. This test is also being used successfully in our laboratory to assess physiological adaptations resulting from exercise therapy in an ongoing breast cancer trial [91].

##### Quality of life

The Functional Assessment of Cancer Therapy-General (FACT-G) and (FACT-B) [99] will be used to assess quality of life. Quality of life is an important clinical outcome and the FACT-G and FACT-B collectively measure five aspects of quality of life: physical, functional, emotional, social and additional concerns specific to breast cancer. Quality of life will be assessed in the week before and after the intervention period and at the mid-intervention time point of 12 weeks.

##### Physical activity behaviour

Physical activity behaviour at the baseline, and at weeks 6,12,18 and 24 will be assessed using the Stanford Seven-Day Physical Activity Recall (PAR) [100], which has been used successfully in a number of studies. This will be administered by telephone in the control patients.

#### Biomarkers associated with disease recurrence and physiological health status

##### Blood sampling

Blood samples will be drawn from an antecubital vein between 8:30–10 a.m. in the morning, following a 12 hour overnight fast. Blood will be collected into Vacutainer tubes (BD Biosciences Ltd, Oxford, U.K.), containing EDTA for leukocyte counts, plasma biomarker and immune function analysis. NK cells and T lymphocytes will be isolated from whole blood within 2 h of venepuncture for analysis of NK Cell and T cell function. Plasma samples will be stored at -80°C for batch-analysis at a later date. All flow cytometric analyses, leukocyte functional assays and enzyme immunoassays will be performed in the Biomedical Sciences Research Centre, Sheffield Hallam University, under the supervision of one of the key personnel, Professor Nicola Woodroofe.

##### Estrogen, stress hormones and inflammatory markers

Plasma IL-6, epinephrine and norepinephrine levels will be measured using commercially available enzyme-linked immunoassays (ELISA) kits (Bio-Quant Inc and Immunobiological Laboratories, Hamburg, R & D Systems UK) and plasma hs-CRP will be measured by the Immunology Department, Northern General Hospital, Sheffield using a turbidimetric assay. Serum estradiol, estrone and estrone sulphate will be measured using high-sensitivity ELISA kits Bio-Quant Inc., CA, USA and Diagnostic Systems Laboratories Inc., OBI-DSL, Oxon, UK). Salivary cortisol levels will be measured on each of three consecutive days at 0800, 1200, 1700 and 2100 hours and the slope of diurnal cortisol variation will be determined from the pooled saliva samples at each daily time point using a regression of the log-transformed cortisol concentrations on sample collection time [38]. Easy-Reach Radio Pagers (British Telecommunications Plc, UK) will be used to signal saliva collection times and the importance of collecting samples at the designated times and recording the actual sample collection time will be emphasized. Saliva samples will be refrigerated, before collection within 2–4 days and frozen at -70°C until analysis[40] using a high sensitivity salivary cortisol enzyme immunoassay (Salimetrics LLC, PA, USA).

##### Differential leukocyte counts

Differential leukocyte counts and other haematological parameters will be measured within 6 hours of blood sampling in the Department of Haematology, Royal Hallamshire Hospital (CPA accredited) using a Coulter STKS automated haematological analyser (Beckman Coulter U.K. Ltd, High Wycomb, U.K.) which is calibrated on a daily basis.

##### Lymphocyte phenotyping

The proportion of NK (CD3^-^CD16^+^CD56^+^) and T cell subsets (CD3^+^CD4^+^; CD3^+^CD8^+^) will be measured using whole blood multicolour flow cytometry. Whole blood will be collected into EDTA anticoagulant and 100 μl aliquots incubated with appropriate combinations of fluorochrome (PE/FITC/PerCP)-conjugated monoclonal antibodies (mAb) to CD3, CD4, CD8, CD16 and CD56 using triple antibody sets (BD Biosciences Ltd). Lymphocyte and monocyte populations will be located on the basis of forward and side scatter and dead cells will be excluded. The proportion of cells expressing the given combination of antigens will be determined using a BD Biosciences FACSort™ flow cytometer and CELLQuest Pro™ data acquisition and analysis software. Appropriate isotype matched, negative control antibodies will be included in all analyses.

##### NK cell activity

NK cell activity will be determined as the capacity of peripheral blood mononuclear cells to kill human erythroleukemia K562 target cells using flow cytometry, essentially as described previously [101]. Briefly, K562 cells in the log phase of growth will be harvested, washed and stained with the stable membrane dye PKH2 (BD Biosciences). Human peripheral blood mononuclear cells will be isolated by density gradient centrifugation on Ficoll (Sigma UK) and monocytes/macrophages will be removed by plastic adherence (37°C, 1 h). Two-fold serial dilutions of effector cells (800 μl; starting at 10^6 ^cells/ml) will be placed in 12 × 75 mm polystyrene tubes with 100 μl PKH2 labelled target cells (10^5 ^cells) to give a range of effector to target cell ratios (80:1 to 5:1). Samples will be incubated at 37°C for 4 h after which time the DNA stain propidium iodide (which only stains non-viable cells) will be added. The proportion of PKH2^+^PI^+ ^(non-viable) K562 cells at each effector to target cell ratio will be determined after 15 min by flow cytometry. Appropriate controls (effector cells only; target cells only) will be included in all experiments.

##### Lymphocyte proliferation assays

Peripheral blood lymphocyte proliferative responses to recall antigen (purified protein derivative, PPD) will be determined. Peripheral blood mononuclear cells will be isolated as described above and 10^5 ^cells incubated for 7 days (37°C, 5% CO_2_) with an appropriate concentration of PPD (~1 μg/ml) in 96-well microtitre plates. Antigen non-specific proliferation will be determined by incubating cells with plate-bound anti-CD3 mAb and soluble anti-CD28 mAb (both 10 μg/ml) (BD Biosciences). For both assays, proliferation will be determined by preincubating cells with 5,6-carboxyfluorescein diacetate succimidyl ester (CFSE)(Molecular Probes) and subsequent measurement of the amount of dye per cell by flow cytometry [102].

### Data Analysis

A mixed factorial ANOVA will be used to assess (i) changes from baseline on all outcome measures following the intervention within patient groups, and (ii) differences in outcome measures between patients in the lifestyle intervention and control groups post intervention. Pearson product moment correlation coefficients (r) will be calculated to determine bivariate relationships among changes in the variables. Statistical significance will be set at *p *< 0.05. All data will be analysed using the SPSS statistical package (SPSS UK Ltd, Woking, U.K.).

## Competing interests

The author(s) declare that they have no competing interests.

## Authors' contributions

JS conceived and drafted the research proposal and sought funding for the study. He is responsible for overall project management. AD contributed to the study design and intervention in and has a role in collation of the psychological data and its analysis. RC contributed intellectual input to the research protocol and is involved with recruitment of volunteers and the provision of medical clearance for patients to participate in the study. NW provided expertise on the biomarker analysis and has a role in overlooking this work. HP provided valuable input to the design of the nutritional intervention and has a role in overlooking this component of the research. NM provided expertise on the quality of life outcomes and has a role in the analysis and interpretation of the quality of life data. VS advised on the practicalities of administering the dietary component of the intervention and has a role in overlooking this component of the research. HC contributed useful knowledge in relation to the exercise intervention and has a role in the hands-on delivery of the research.

## Pre-publication history

The pre-publication history for this paper can be accessed here:


